# Shotgun-induced rupture leading to subcutaneous hydatidosis: A rare complication of hepatic echinococcosis

**DOI:** 10.1016/j.radcr.2025.08.077

**Published:** 2025-09-22

**Authors:** Reza jalli, Seyed Sina Dehghani, Seyedeh Nadia Tabatabaeifar, Sedighe hooshmandi

**Affiliations:** a Medical Imaging Research Center, Shiraz University of Medical Sciences, Shiraz, Iran; b Department of Radiology, Shiraz University of Medical Sciences, Shiraz, Iran; c Internal Medicine Department, School of Medicine, Shiraz University of Medical Sciences, Shiraz, Iran; d Medical Imaging Research Center Department of Radiology, Shiraz University of Medical Sciences, Shiraz, Iran

**Keywords:** Subcutaneous hydatidosis, Cystic echinococcosis

## Abstract

Hydatid disease, caused by Echinococcus granulosus, typically affects the liver and lungs, with subcutaneous involvement being exceedingly rare. We report a unique case of a 53-year-old man with a history of chronic obstructive pulmonary disease (COPD) and a prior shotgun injury, presenting with dyspnea and COPD exacerbation. An abdominopelvic CT scan revealed multiple hepatic hydatid cysts and an unusual subcutaneous multilocular cyst in the right hemithorax, likely secondary to traumatic rupture from the shotgun injury. This case highlights the diagnostic utility of CT in identifying atypical hydatid cyst presentations and underscores the rare phenomenon of subcutaneous dissemination following trauma. We discuss the imaging findings, clinical implications, and management strategies, emphasizing the importance of considering hydatidosis in differential diagnoses of cystic lesions in endemic regions.

## Introduction

Hydatid disease, or cystic echinococcosis, is a zoonotic infection caused by the larval stage of Echinococcus granulosus, commonly transmitted via the fecal-oral route in endemic regions such as the Mediterranean, Middle East, and parts of South America [1]. The liver is the most frequently affected organ (75%), followed by the lungs (15%), with rare involvement of other sites including subcutaneous tissue [2]. Subcutaneous hydatid cysts are exceptionally uncommon, accounting for only 1%-5.4% of cases, and are typically secondary to iatrogenic spillage or spontaneous rupture of visceral cysts. Primary subcutaneous hydatidosis without visceral involvement is even rarer, with few documented cases [3]. Computed tomography (CT) is a cornerstone in diagnosing hydatid cysts, particularly when ultrasound is inconclusive or when assessing complications such as rupture or calcification [4]. On CT, hydatid cysts typically appear as well-defined, non-enhancing cystic lesions with internal features like undulating membranes, daughter cysts, or calcifications, aiding differentiation from other cystic entities [5]. Subcutaneous hydatid cysts present a diagnostic challenge due to their atypical location, often mimicking soft tissue masses or abscesses, necessitating correlation with clinical history and serological tests [6]. We present a rare case of subcutaneous hydatidosis likely resulting from traumatic rupture of a hepatic cyst following a shotgun injury, illustrating the role of CT in uncovering such unusual presentations.

## Case presentation

**Fig. 1:**
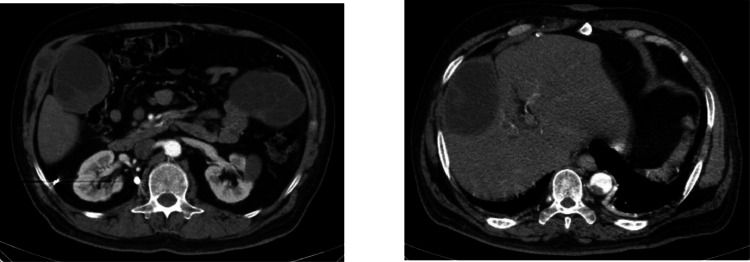
The CT revealed a normal-sized liver with a subcapsular, cystic structure in segment VIII measuring 98 × 65 mm, containing internal undulating membranes suggestive of a hydatid cyst. A similar cystic lesion measuring 93 × 61 mm was identified adjacent to segment VI, also consistent with hydatidosis. Further scrutiny identified a cystic structure in the left upper quadrant measuring 112 × 85 mm, with internal membranes and daughter cysts, suggestive of another hydatid cyst. There is evidence of a hyperdense suspiciously metallic foreign body adjacent to right side psoas muscle in perirenal space at L1 level.

**Fig. 2:**
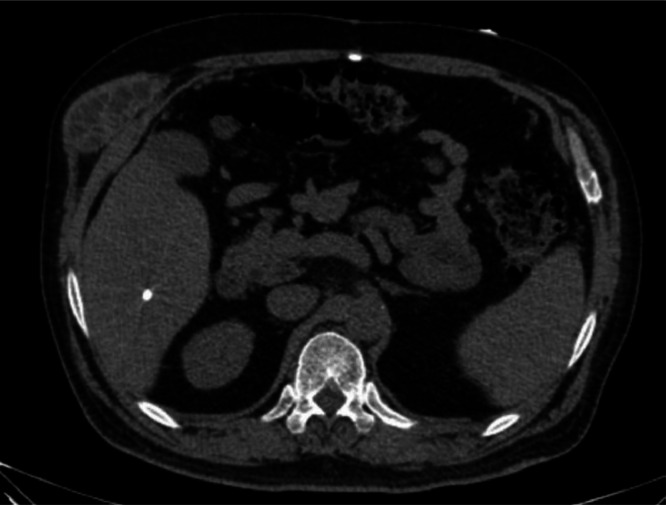
Additional findings included tiny calcified foci in the right hepatic lobe, suggestive of metallic fragments from the prior injury.

**Fig. 3:**
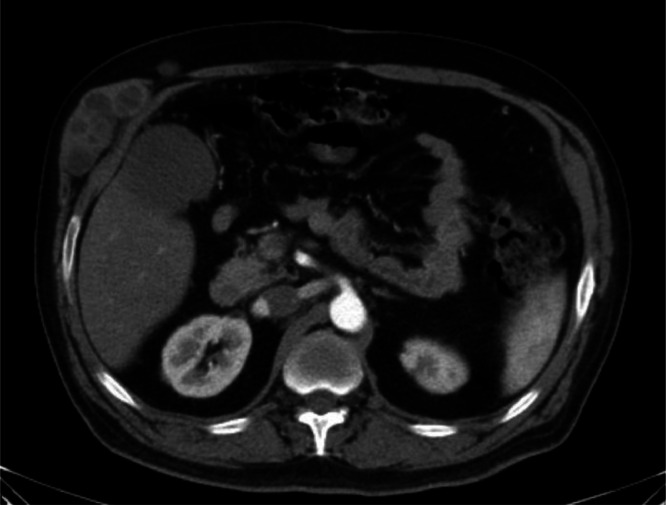
Most strikingly, a multilocular subcutaneous cystic lesion measuring 55 × 28 mm was observed in the lower right hemithorax, raising suspicion of a subcutaneous hydatid cyst.

**Fig 4:**
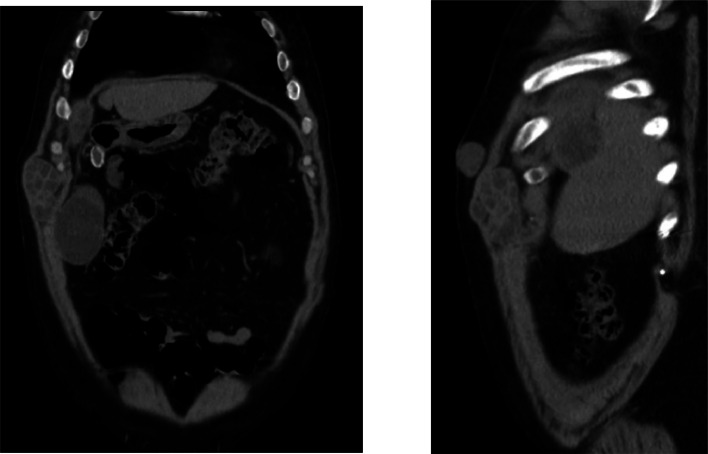
Coronal and sagittal Ct scan revealed the mentioned subcutaneous hydatid cyst.

A 53-year-old male with a known history of chronic obstructive pulmonary disease (COPD) presented to our hospital with worsening dyspnea and symptoms consistent with a COPD exacerbation. He was a lifelong resident of a rural area endemic for hydatid disease and had a history of a shotgun injury to the abdomen years prior (with no history of hydatid disease diagnosed at that time). There was no recent travel outside the endemic region. His medical history included a shotgun injury several years prior, with retained foreign bodies noted in the abdominal region. According to the patient’s medical history, he was asymptomatic prior to the shotgun injury despite the presence of hepatic lesions, and no palpable masses were noted on the right flank. Approximately 2 months after the trauma, he developed a gradually enlarging swelling on the right side of the abdomen, which was confirmed on physical examination by the attending physician, leading to referral to a specialized hospital for further diagnostic evaluation and management. As part of the evaluation a contrast-enhanced CT scan of the chest and abdomen was obtained. The CT scan revealed no acute pulmonary embolus but showed hyperinflated lungs consistent with COPD. Incidentally, multiple cystic lesions were noted in the abdominal and chest regions. The combination of imaging findings strongly suggested disseminated hydatid disease involving the liver, peritoneal/left upper quadrant region, and the subcutaneous tissue of the chest wall. The subcutaneous lesion in the chest wall was presumed to represent a secondary hydatid cyst, possibly resulting from spillage of a hepatic cyst due to the past trauma. Serological testing for hydatid disease (indirect hemagglutination and ELISA) was performed and later returned positive for Echinococcus antibodies, supporting the imaging diagnosis Fig. 1, Fig. 2, Fig. 3, Fig 4.

Given the patient’s history of a shotgun injury, we hypothesized that trauma-induced rupture of a hepatic cyst may have seeded the subcutaneous tissue.

The patient was prescribed albendazole at a dose of 400 mg twice daily for an adult, administered in cycles of 28 days with a 14-day drug-free interval. After completion of the initial treatment course, the surgical team reassessed the patient to determine whether surgical intervention was indicated based on clinical and imaging findings during follow-up.

## Discussion

Hydatid disease typically manifests in the liver and lungs, with hepatic involvement occurring in up to 75% of cases due to portal venous filtration of Echinococcus larvae [1]. Subcutaneous hydatid cysts are rare, with an incidence of 1%-5.4% among all hydatidosis cases, and are usually secondary to visceral cyst rupture or surgical spillage. Primary subcutaneous hydatidosis, without visceral involvement, is exceptionally rare and poorly understood, possibly arising from direct larval implantation through skin breaches [3]. Our case, however, suggests a traumatic etiology, where a shotgun injury likely caused hepatic cyst rupture, disseminating cyst contents into the subcutaneous plane. CT imaging is pivotal in diagnosing hydatid cysts, offering high sensitivity for detecting characteristic features such as non-enhancing cystic structures, internal membranes, daughter cysts, and calcifications [4]. In our patient, the hepatic cysts displayed classic signs—undulating membranes and daughter cysts—while the subcutaneous lesion’s multilocular appearance aligned with reported imaging findings of soft tissue hydatidosis [5]. The presence of metallic foreign bodies and subcutaneous hyperdense foci from the shotgun injury provided a plausible mechanism for cyst rupture, a complication reported in only a handful of cases. Unlike spontaneous or iatrogenic rupture, trauma-induced dissemination to subcutaneous tissue is extraordinarily rare, making this case noteworthy. Differential diagnoses for subcutaneous cystic lesions include abscesses, hematomas, and neoplasms, but the multilocular nature and endemic context favored hydatidosis [6]. Serological confirmation and histopathology solidified the diagnosis, consistent with guidelines recommending combined imaging and serology for atypical presentations [7]. Management involved surgical excision of the subcutaneous cyst and percutaneous aspiration of hepatic cysts with albendazole therapy, aligning with evidence supporting complete cyst removal to prevent recurrence [8]. The absence of recurrence at follow-up supports this approach, though long-term monitoring is essential given the risk of dissemination [9]. This case underscores the diagnostic prowess of CT in unveiling rare complications of hydatid disease and highlights trauma as a potential trigger for subcutaneous spread. Clinicians in endemic regions should consider hydatidosis in patients with cystic lesions and a history of trauma, even in atypical locations.

## Conclusion

We report a rare instance of subcutaneous hydatidosis likely secondary to traumatic rupture of a hepatic cyst following a shotgun injury, diagnosed via CT in a 53-year-old man with COPD. This case illustrates the critical role of CT in identifying atypical hydatid cyst presentations and emphasizes the need for a high index of suspicion in endemic areas, particularly with a trauma history. Successful management with surgery and albendazole highlights the efficacy of combined approaches in such rare scenarios.

## Ethical considerations

Written informed consent was obtained from patient for the publication of this case report, including the use of accompanying images. This report complies with the ethical standards set forth in the Declaration of Helsinki.

## Patient consent

Written informed consent was obtained from the patient for publication of this case report and accompanying images. A copy of the written consent is available for review by the Editor-in-Chief of this journal on request.
